# Identifying complementary and alternative medicine recommendations for anxiety treatment and care: a systematic review and critical assessment of comprehensive clinical practice guidelines

**DOI:** 10.3389/fpsyt.2023.1290580

**Published:** 2023-12-12

**Authors:** Fei-Yi Zhao, Gerard A. Kennedy, Peijie Xu, Russell Conduit, Yan-Mei Wang, Wen-Jing Zhang, Hui-Ru Wang, Li-Ping Yue, Yu-Ling Huang, Yin Wang, Yan Xu, Qiang-Qiang Fu, Zhen Zheng

**Affiliations:** ^1^Department of Nursing, School of International Medical Technology, Shanghai Sanda University, Shanghai, China; ^2^Shanghai Municipal Hospital of Traditional Chinese Medicine, Shanghai University of Traditional Chinese Medicine, Shanghai, China; ^3^Institute of Health and Wellbeing, Federation University, Mount Helen, VIC, Australia; ^4^School of Health and Biomedical Sciences, RMIT University, Bundoora, VIC, Australia; ^5^Institute for Breathing and Sleep, Austin Health, Heidelberg, VIC, Australia; ^6^School of Computing Technologies, RMIT University, Melbourne, VIC, Australia; ^7^Yangpu Hospital, School of Medicine, Tongji University, Shanghai, China

**Keywords:** complementary and alternative medicine, complementary and integrative health, anxiety, herbalism, mind–body practice, clinical practice guidelines, guidelines, systematic review

## Abstract

**Background:**

Clinical practice guidelines (CPGs) are used to guide decision-making, especially regarding complementary and alternative medicine (CAM) therapies that are unfamiliar to orthodox healthcare providers. This systematic review aimed to critically review and summarise CAM recommendations associated with anxiety management included in the existing CPGs.

**Methods:**

Seven databases, websites of six international guidelines developing institutions, and the *National Centre for Complementary and Integrative Health* website were systematically searched. Their reporting and methodological quality were evaluated using the *Reporting Items for practice Guidelines in Healthcare* checklist and the *Appraisal of Guidelines for Research and Evaluation* (2nd version) instrument, respectively.

**Results:**

Ten CPGs were included, with reporting rates between 51.4 and 88.6%. Seven of these were of moderate to high methodological quality. Seventeen CAM modalities were implicated, involving phytotherapeutics, mind–body practice, art therapy, and homeopathy. Applied relaxation was included in 70% CPGs, which varied in degree of support for its use in the treatment of generalised anxiety disorder. There were few recommendations for other therapies/products. Light therapy was not recommended for use in generalised anxiety disorder, and *St John’s wort* and mindfulness were not recommended for use in social anxiety disorder in individual guidelines. Recommendations for the applicability of other therapies/products for treating a specific anxiety disorder were commonly graded as “unclear, unambiguous, or uncertain”. No CAM recommendations were provided for separation anxiety disorder, specific phobia or selective mutism.

**Conclusion:**

Available guidelines are limited in providing logically explained graded CAM recommendations for anxiety treatment and care. A lack of high-quality evidence and multidisciplinary consultation during the guideline development are two major reasons. High quality and reliable clinical evidence and the engagement of a range of interdisciplinary stakeholders are needed for future CPG development and updating.

**Systematic review registration:**

https://www.crd.york.ac.uk/prospero/display_record.php?ID=CRD42022373694, identifier CRD42022373694.

## 1 Background

Anxiety disorders, as a collective entity, are pervasive and include discrete diagnoses of social anxiety disorder, separation anxiety disorder, generalised anxiety disorder, panic disorder, specific phobia, agoraphobia, and selective mutism (1). Up to 33.7% of the population is affected by at least one anxiety disorder in their lifetime (2). Anxiety disorders often cause clinically significant functional impairment, distress, and/or disability risk (3, 4). They are associated with cardiovascular disease, gastrointestinal problems, migraine, genitourinary difficulties (5), stroke, and cognitive decline (4). Social anxiety disorders also emerge as a unique risk factor for the onset of alcohol (6), cannabis (6), and nicotine dependence (7). Anxiety disorders accounted for 390 disability-adjusted life years per 100,000 persons globally in 2010 (8). Both cross-sectional community (9, 10) and clinical (11, 12) studies show in univariate models that anxiety disorders are associated with suicidal ideation, suicide attempts and/or completed suicides. Due to the loss of productivity and earnings (indirect costs) and high medical resource use (direct costs), anxiety disorders contribute considerably to economic costs (13). In North America and Europe, patients with generalised anxiety disorder had significantly higher annual median medical costs than those without generalised anxiety disorder (US $2,375 Vs. US $1448) (14). The total cost of anxiety disorders in Japan in 2008 reached US $ 20.5 billion (15). In UK, anxiety disorders are the fifth most costly neurological and psychiatric disorders with a cost of €11,687 million *per annum* (16). These economic costs might still be underestimated, given anxiety disorders frequently go under-diagnosed and under-treated in primary care settings for a variety of reasons (e.g., a focus on somatic symptoms, the stigma of mental illness, confounding symptoms caused by comorbid depression, etc.) (17, 18).

Cognitive behavioural therapy (CBT) has been demonstrated to be more effective than other psychosocial therapies in the treatment of anxiety disorders (19). However, its overall treatment response rates across anxiety disorders only averaged 49.5% at post-treatment and 53.6% at follow-up (19). Access to and high cost of CBT are issues as well (20). Pharmacology is another treatment strategy for anxiety disorders (20). Benzodiazepines are efficacious in most anxiety disorders; selective serotonin reuptake inhibitors and serotonin-noradrenaline reuptake inhibitors show mild to moderate positive effects in generalised anxiety disorder, social anxiety disorder, agoraphobia and panic disorder (21). Their tolerance, dependence, adverse effects (e.g., sexual dysfunction and weight gain, etc.), relatively slow onset of action, and withdrawal reactions on termination, however, can be major deterrents to compliance, and affect over 50% of users in the longer-term (20, 22, 23). Complementary and alternative medicine (CAM) therapies for anxiety also have many proponents. In a cross-sectional and longitudinal survey covering 1,004 adults who met DSM-IV criteria for social anxiety disorder, generalised anxiety disorder, panic disorder, or post-traumatic stress disorder in the United States, 42.8% of respondents reported the use of a variety of CAM treatments, such as supplements, herbal medicine, or relaxation (24). In Tanzania, 20.5% of patients diagnosed with anxiety disorders and 27.8% of patients diagnosed with mixed anxiety-depressive disorder sought help from the traditional healers (25). In an Australian cross-sectional survey, 72.8% of interviewees reported having used herbal medicines, such as *Chamomile*, *Lavender*, or *Valerian* to manage anxiety symptoms in their lifetime (26).

Given the interest in CAM is increasing, evidence-informed guidance is required to assist patients and healthcare providers to make adequately informed decisions regarding utilisation of CAM therapies (27). Clinical practice guidelines (CPGs) serve a crucial purpose in assisting clinicians to access critically-evaluated evidence-based recommendations for the care of patients (28). Orthodox healthcare professionals, particularly in western world, are less exposed to CAM knowledge in their medical education and training (29). Consequently, CPGs are generally relied on as an evidence-based framework to understand whether the use of a CAM modality is reasonable, and a basis for informed and shared decision-making with patients about associated risks and/or benefits (29). In accordance with the available literature, many CAM approaches such as *Kava* (30), yoga (31), mindfulness-based meditation (31), and acupuncture (32) have shown anxiolytic potential. Therefore, the question remains whether these therapies have been integrated into CPGs and recommended to clinical professionals for anxiety management. Furthermore, are there CAM therapies that have been explicitly judged to be ineffective or harmful? Finally, what is the strength of these CAM recommendations? Bridging these knowledge gaps is of crucial clinical importance and is the purpose of our present study.

## 2 Materials and methods

### 2.1 Registration and eligibility criteria

The current review followed the *Preferred Reporting Items for Systematic Reviews and Meta-Analyses (PRISMA) 2020 Statement* guidelines (33). A protocol was prospectively registered with PROSPERO (Identifier: CRD42022373694). Only formally published guidelines addressing anxiety disorders and containing CAM recommendations for anxiety treatment and/or care were included in the present review. The type of anxiety disorders was not limited. Whereas, CPGs related to obsessive-compulsive disorder or post-traumatic stress disorder were not included as these two disorders had been removed from the category of anxiety disorders in the DSM-V (34). In the current study, the specific modality and attributes of each CAM therapy were based on the classification updated by the *US National Centre for Complementary and Integrative Health* (NCCIH) (35). Briefly, CAM approaches were generally classified into five categories by their primary therapeutic input: nutritional, psychological, physical, combinations (e.g., combined psychological and physical, or combined psychological and nutritional, etc.) and other complementary health approaches (Appendix 1). The language of the guidelines was restricted to English or Chinese, while the publication date was not restricted. Seeing that this review aimed to capture accessible CAM recommendations for orthodox medical professionals, only comprehensive CPGs were considered. Specialized CAM guidelines (e.g., Ayurveda, herbalism, homeopathic, acupuncture guidelines, etc.) were excluded from the analysis. The guidelines were also excluded if they: (1) did not include a systematic literature search process; (2) did not clearly describe the systems or methods used for grading the evidence and recommendations; (3) protocol, translation, or interpretation of CPGs; and/or (4) earlier versions of CPGs, for which an updated version is available.

### 2.2 Data sources and searches

A thorough search was undertaken using three English electronic databases [MEDLINE (via PubMed), AMED: Allied and Complementary Medicine Database, and EMBASE (via OVID)] and four Chinese electronic databases [China biomedical literature service system (SinoMed), Wanfang database, Chongqing VIP database (CQVIP), and China National Knowledge Infrastructure (CNKI)] to identify relevant CPGs published before July 2023. The search strategies, as outlined in Appendix 2, included indexed headings and keywords commonly used in the literature to describe anxiety disorders and guidelines. To achieve literature saturation and gather a wide range of relevant sources, reference tracking of all included CPGs was conducted. In addition, we manually searched a compiled list of CAM CPGs provided by NCCIH website and six professional websites of international guideline developing institutions (Appendix 3).

### 2.3 Selection of guidelines and data extraction

The literature retrieved was imported into the *Rayyan* (36), and then was initially screened by two experienced investigators (LP-Y and YM-W) checking titles and abstracts via this software. *Rayyan* was also used to automatically identify duplicate literature. When titles and abstracts implied that a guideline was potentially eligible for inclusion, the full guideline was obtained and further cross-checked for inclusion (Y-X and PJ-X). Two purpose-designed spreadsheet were adopted to extract the following data from each guideline: first author, year of publication and region of development, primary developer/publishing entity, basis for recommendations formation (evidence-based or both evidence- and consensus-based), types of anxiety disorders discussed in the guideline and the diagnostic criteria on which they are based (e.g., DSM-V and ICD-10, etc.), information related to systematic search and grants, rating system for the quality of evidence and strength of recommendations, and the modalities of CAM included. In each guideline, the type of anxiety disorder targeted by CAM recommendations and the propensity of these recommendations (supported or not supported) were also extracted and plotted visually.

### 2.4 Methodological and reporting quality appraisal

Four investigators (WJ-Z, YL-H, YM-W, and HR-W) independently evaluated the methodological quality of each selected CPG using the Appraisal of Guidelines Research and Evaluation (2nd version) (*AGREE II*) instrument (37). To understand the reporting quality, another two investigators (Y-W and PJ-X) independently appraised the compliance of each CPG to the Reporting Items for practice Guidelines in Healthcare (*RIGHT*) checklist (38) after assessing the content reported in the guideline.

The *AGREE II* is 23-item instrument that addresses six guideline quality-related domains, namely *Scope and purpose*; *Stakeholder involvement*; *Rigor of development*; *Clarity of presentation*; *Applicability*; and *Editorial independence*. Each item was assigned a score ranging from seven to one depending on how much they agree or disagree that the guideline conforms with the provided criteria (7 = strongly agree, 1 = strongly disagree). Domain scores were calculated by dividing the difference between the obtained score and the maximum possible score by the difference between the minimum and the maximum possible score. The standardized scores range from 0% (the minimum) to 100% (the maximum) (39). A previous study suggested that to reflect the overall score of a CPG, the global score could be obtained by calculating the sum of the six domain scores and dividing by 600%, with a global score ranging from 0 to 100% (40).

The *RIGHT* checklist comprises 35 items organized into seven domains: *Basic information*; *Background*; *Evidence*; *Recommendations*; *Review and quality assurance*; *Funding, declaration and management of interests*; and *Other information*. Each item was assigned a dichotomous score of “Yes (majority of the relevant information was reported)"or “No (relevant information was not reported)” (38). Discrepancy was resolved through discussion by the raters. The number of reported items of each CPG was documented and then translated into a reporting rate (0 to 100%).

Training and pilot tests were organized before the appraisal practice to ensure all investigators had a clear understanding of each item in the assessment tool and enhance internal agreement. Given the *AGREE II* instrument was rated by four separate investigators, we also performed an intra-class correlation coefficients (*ICCs*) consistency analysis to calculate the *Kappa* value for this evaluation using IBM SPSS Statistics 29. The strength of agreement for *ICC* point estimates was assessed as poor (0.01–0.20), fair (0.21–0.40), moderate (0.41–0.60), good (0.61–0.80), or excellent (0.81–1.00) (41).

### 2.5 Data synthesis and quality grading

For each guideline, the scores assigned to each domain of the *AGREE II* instrument and the reporting rate in each domain of the *RIGHT* checklist were calculated, and then presented as a stacked polar chart and a clustered bar chart, respectively.

We also constructed a bubble plot to show the overall quality of each included guideline, with the *Y*-axis denoting the global scores of the *AGREE II* instrument and *X*-axis denoting the average reporting rate of the *RIGHT* checklist. Accordingly, all included guidelines were divided into three clusters: low-quality CPG (*X* value <60 and *Y* value <50), moderate-quality CPG (60 ≤ *X* value <80 and 50 ≤ *Y* value <70), or high-quality CPG (80 ≤ *X* value and 70 ≤ *Y* value). The three colored spheres, namely red (low quality), yellow (moderate quality), and green (high quality), were employed to distinguish and visualize the overall quality of each guideline. The bubble plot allowed for a summary and analysis of the relatively reliability and applicability of CAM recommendations derived from the guidelines. Referring to a previous study with the same design (42), the high-, moderate- and low-quality guidelines visualised in the bubble plot were suggested as “recommended,” “recommended with modifications,” and “not recommended,” respectively.

## 3 Results analysis

### 3.1 Guidelines screening

**Figure 1:**
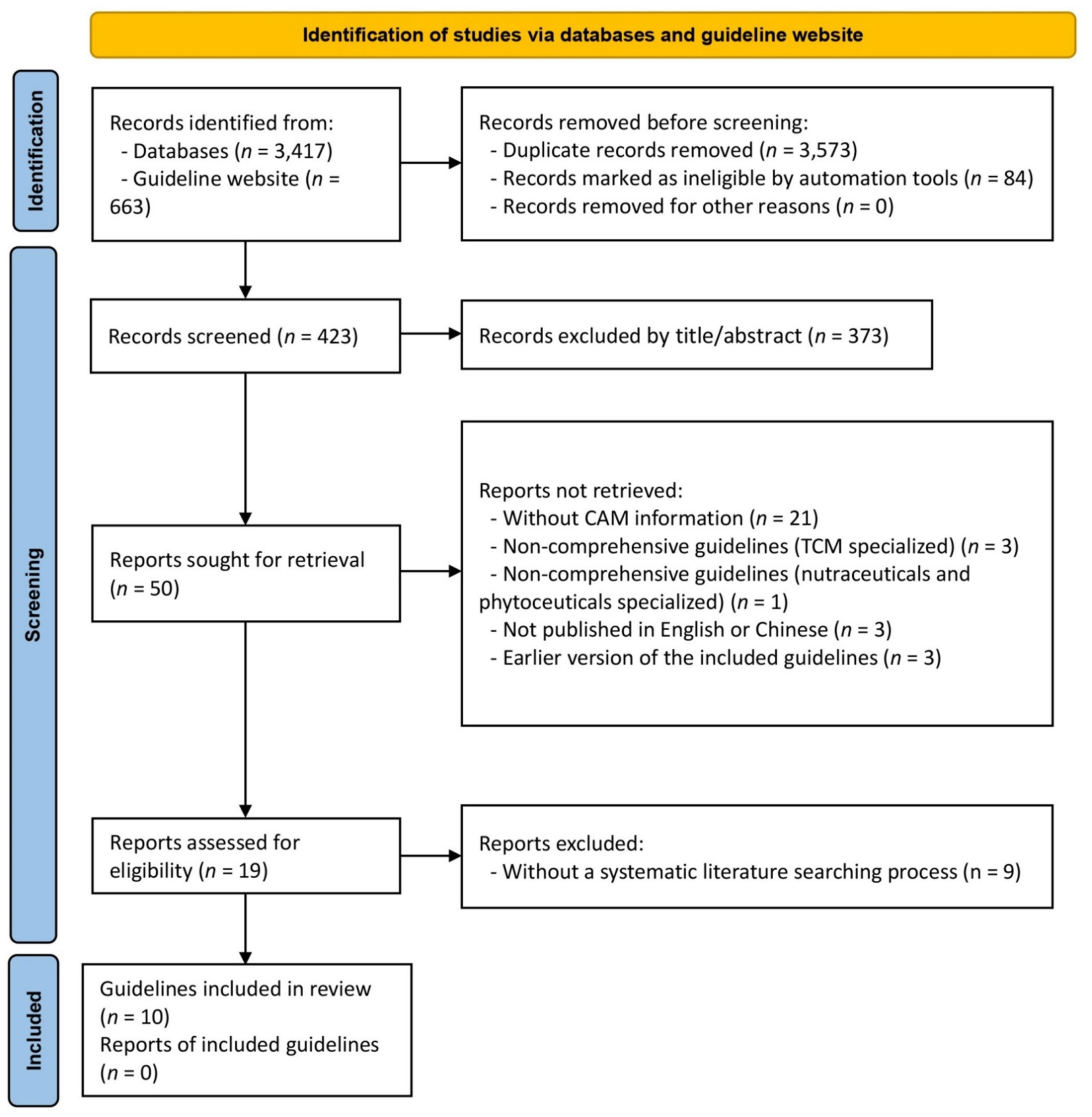
Flow diagram of the study selection process.

The database searches initially retrieved 4,080 works which were reduced to 423 after removal of duplicates and exclusion of irrelevant records by title and abstract. The full text of a further 50 guidelines were compared against the inclusion and exclusion criteria and another 40 guidelines were rejected, leaving ten guidelines for inclusion in the review (Figure 1). All these guidelines were published in English. A list of discarded 40 guidelines and detailed reasons for exclusion is provided in Appendix 4.

### 3.2 Guidelines characteristics

Table 1 summarized the features of the ten included CPGs. Eligible guidelines were published from 2003 to 2022, in the UK (*n* = 3), Canada (*n* = 2), Australia & New Zealand (*n* = 2), United States (*n* = 1), Germany (*n* = 1), or Spain (*n* = 1). Sixty percent of these CPGs were the updated versions (43–48).

**Table 1:** Characteristics of the eligible clinical practice guidelines.

Author, year	Evidence-based (EB), or consensus-based (CB)	Population	Types of anxiety disorders (Diagnosis)	Country	Primary developer/ Publishing entity	Version	Systematical search included	Databases	Search strategies	Search year	Funding	CAM modalities included
Andrews et al. 2018 (43)	Both EB & CB	General	GAD, PD, SAD (DSM-V)	Australia & New Zealand	RANZCP	Updated	Yes	Cochrane, MEDLINE, EMBASE, PsycINFO	Yes	Inception – Dec 2017	Funding from RANZCP	Applied relaxation, mindfulness
Bandelow et al. 2022 (44)	Both EB & CB	Adults (≥ 18)	GAD, PD with or without agoraphobia, SAD, SP (ICD-10)	Germany	ASMS	Updated	Yes	PubMed, WOS	Yes	Sept 2013 – Jun 2019	Open access funding enabled and organized by Projekt DEAL	Applied relaxation, homeopathy, music/dance/art therapy, yoga
Greenlee et al. 2017 (45)	EB	Breast cancer survivors	GAD (NR)	USA	SIO	Updated	Yes	CINAHL, MEDLINE, EMBASE, PsycINFO	NR	1990–2015	None	Acupuncture, applied relaxation, massage, meditation, music therapy, yoga
NCCMH 2011a (46)	Both EB & CB	General	GAD, PD, SAD, SP (DSM-IV)	UK	NCCMH	Updated	Yes	CDSR, CENTRAL, CINAHL, DARE, EMBASE, MEDLINE, PsycINFO	Yes	Inception – Sept 2010	Funding from NICE	Applied relaxation
NCCMH 2011b (47)	Both EB & CB	Adults	GAD (DSM-IV)	UK	NCCMH	Updated	Yes	AMED, CDSR, CENTRAL, CINAHL, DARE, EMBASE, HTA database, IBSS, MEDLINE, PsycINFO	Yes	Inception – Nov 2009	Funding from NICE	Acupuncture, applied relaxation, *Chamomile*, *Galphimia Glauca*, *Ginkgo Biloba*, hypnosis, *Passiflora*, *Silexan*, *Valerian*
NCCMH 2013 (48)	Both EB & CB	General	SAD (NR)	UK	NCCMH	Updated	Yes	Cochrane, MEDLINE, Pubmed, PsycINFO	NR	Inception – 2015	Funding from NICE	Mindfulness, *St John’s wort*
Howell et al. 2015 (49)	Both EB & CB	Cancer survivors	GAD (DSM-V-TR)	Canada	CAPO & CPAC	Original	Yes	CDSR, CENTRAL, CINAHL, EMBASE, MEDLINE, PsychINFO	Yes	2009 – May 2015	Health Canada	Aromatherapy massage
Katzman et al. 2014 (50)	Both EB & CB	General	Agoraphobia, GAD, PD, SAD, SP, separation anxiety disorder (DSM-IV)	Canada	CAGIG	Original	Yes	MEDLINE, PsycINFO	NR	1980–2012	Unrestricted educational grants from several companies	Acupuncture, *Galphimia Glauca*, light therapy, meditation, *Passiflora*, *Silexan* (*Lavender* oil), *St John’s wort*, *Valerian*, yoga
Hurtado et al. 2020 (51)	EB	General	GAD (DSM-IV)	Spain	RUHM& DPHCMG	Original	Yes	CINAHL, Cochrane Plus, EMBASE, Índice Médico Español, PsycINFO, PubMed	Yes	NR	None	Applied relaxation
RANZCP 2003 (52)	EB	General	Agoraphobia, PD (DSM-IV)	Australia & New Zealand	RANZCP	Original	Yes	Cochrane, MEDLINE, EMBASE, PsycINFO	Yes	Inception – 1999	NMHS & NZHFA	Applied relaxation, hypnosis

NR, no reports; AMED, Allied and Complementary Medicine Database; ASMS, Association of Scientific Medical Societies (Germany); CAGIG, Canadian Anxiety Guidelines Initiative Group; CAPO, Canadian Association of Psychosocial Oncology; CDSR, Cochrane Database of Systematic Reviews; CENTRAL, Cochrane Central Register of Controlled Trials; CINAHL, Cumulative Index to Nursing and Allied Health Literature; CPAC, Canadian Partnership Against Cancer; DARE, Cochrane Database of Abstracts of Reviews of Effects; DSM-IV, Diagnostic and Statistical Manual of Mental Disorders (Fourth Edition); DSM-V, Diagnostic and Statistical Manual of Mental Disorders (Fifth Edition); DSM-V-TR, DSM-V (Text Revision); EMBASE, Excerpta Medica Database; GAD, generalised anxiety disorder; HTA, Health Technology Assessment; IBSS, International Bibliography of the Social Sciences; ICD-10, International Classification of Diseases (Tenth Edition); MEDLINE, Medical Literature Analysis and Retrieval System Online; NCCMH, National Collaborating Centre for Mental Health; NICE, National Institute for Health and Clinical Excellence; NMHS & NZHFA, National Mental Health Strategy (Australia) and the New Zealand Health Funding Authority; PsycINFO, Psychological Information Database; PD, panic disorder; RANZCP, Royal Australian and New Zealand College of Psychiatrists; RUHM& DPHCMG, Regional University Hospital of Málaga and District of Primary Health Care Málaga-Guadalhorce; SAD, social anxiety disorder; SIO, Society for Integrative Oncology; SP, specific phobia; WOS, Web of Science.

The target populations varied across these guidelines. Classified by pathogenesis, one was designed for anxiety in cancer survivors (49), one was designed for anxiety in only breast cancer survivors (45), and the remaining CPGs were not limited to any particular group. Classified by the sociodemographic characteristics, two CPGs were designed for adults (44, 47), and the remaining guidelines did not limit age.

None of the ten guidelines included all seven types of anxiety. Generalised anxiety disorder was included in eight CPGs, followed by social anxiety disorder in five CPGs, panic disorder with/without agoraphobia in five CPGs, specific phobia in three CPGs, and separation anxiety disorder in one CPG. However, none of these CPGs provided CAM recommendations for specific phobia or separation anxiety disorder. In addition, none of the ten CPGs included discussion of selective mutism (Table 1).

Three of the ten CPGs were developed based on evidence only, and the remaining guidelines were developed based on both evidence and expert consensus. While all CPGs were evidence-based with systematic literature searches, three did not detail the specific search strategies (45, 48, 50); one did not describe the search year used for searching the databases (51).

The ten included CPGs involved a total of five grading systems adopted to quantify the level of evidence and the strength of recommendation. Of these, five CPGs used the *Grading of Recommendations Assessment (GRADE) System*; two CPGs used the *Royal Australian and New Zealand College of Psychiatrists (RANZCP) System*; and the remaining three CPGs used *Association of Scientific Medical Societies (ASMS) System*, *Canadian Anxiety Guidelines Initiative Group (GAGIG) System*, and *U.S. Preventive Services Task Force Grading System (USPSTFGS), respectively* (Table 2).

**Table 2:** Grading systems adopted in the included clinical practice guidelines.

Grading system	Codes of evidence and recommendation		Number of CPGs (%)	CPGs
	Levels of evidence	Strengths of recommendation		
ASMS	Ia, Ib, IIa, IIb, III, IV	A+, B+, 0+, A−, B−, 0−	1 (10.0)	(44)
CAGIG	1, 2, 3, 4	First-line, Second-line, Third-line, Not recommended	1 (10.0)	(50)
GRADE	High, Moderate, Low, Very low	Strong, Weak	5 (50.0)	(46–49, 51)
RANZCP	I, II, III-1, III-2, III-4, IV	Not recommended, consensus-based recommendation, evidence-based recommendation	2 (20.0)	(43, 52)
USPSTFGS	High, Low	A, B, C, D, H, I	1 (10.0)	(45)

ASMS, Association of Scientific Medical Societies (Germany); CAGIG, Canadian Anxiety Guidelines Initiative Group; GRADE, Grading of Recommendations Assessment, Development and Evaluation; RANZCP, Royal Australian and New Zealand College of Psychiatrists; USPSTFGS, U.S. Preventive Servi ces Task Force Grading System.

### 3.3 Quality of CPGs

#### 3.3.1 Methodological quality of CPGs

There was good to excellent inter-rater reliability (*IRR*) across the four investigators in methodological quality appraisal, with the overall *ICCs* statistics varying from 0.70 [95%*CI* (0.48 to 0.84), *p* < 0.01] to 0.90 [95%*CI* (0.82 to 0.95), *p* < 0.01] (Appendix 5).

**Figure 2:**
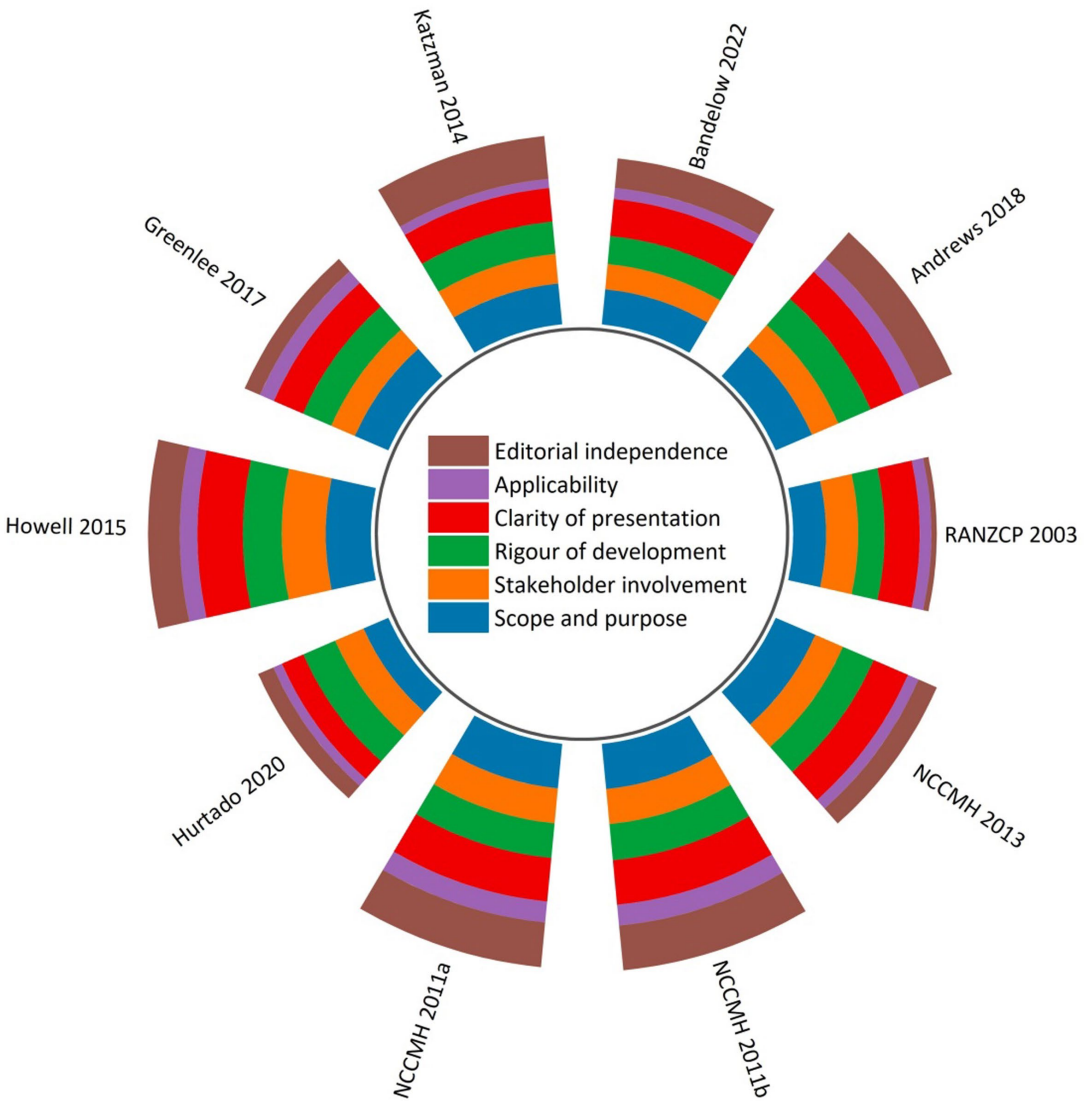
Global *AGREE II* scores by domain across 10 clinical practice guidelines.

Figure 2 and Appendix 6 showed the sum of the *AGREE II* scores of each guideline. Three CPGs were rated as high-quality in methodology (46, 47, 49), four were moderate-quality (45, 51, 52). The remaining three low-quality CPGs were scored less than 30 points for the “*Editorial independence*” domain because of a lack of transparent information in regard to the influence of the funding body on the guideline recommendations and/or the competing interests of guideline development panel members. While CPGs with moderate to high quality included descriptions of competing interests, they rarely reported the approaches utilised to seek competing interests and measures taken to minimize the impacts of competing interests on CPG development or recommendations formulation.

The highest average score appeared in the “*Scope and purpose*” domain, indicating that the overall aims, health questions, and population for whom the guideline is meant to apply were specific and clear except for one guideline that scored less than 60% in this domain (51). This was followed by the “*Clarity of presentation*” domain (69.9% ± 12.8%), which requires the recommendations to be specific and unambiguous, key recommendations to be easily accessible, and different options for management of the condition/health issue to be conspicuously presented.

In respect to scaled domain percentages of CPGs, the “*Applicability*” domain was assigned the lowest average score (27.5% ± 8.6%). Without detailed descriptions of facilitators and barriers to the guideline’ application, direct advice/tools facilitating the translation of recommendations into practice, and/or monitoring and/or auditing criteria, each of the reviewed guidelines scored lower in this domain than they did in the other five domains. Only two CPGs relatively adequately addressed the resource implications in the recommendations application (46, 47).

The “Rigor of development” (62.8% ± 7.2%) and the “Stakeholder involvement” (60.1% ± 10.3%) were two domains with scores slightly beyond the average scores of all six domains (58.2% ± 10.4%). Only one guideline achieved relatively high scores (>70%) in the “*Rigor of development*” domain due to overall methodological rigor (49). Most guidelines lost scores in item 13 (an external review of the guideline by experts prior to its publication) and item 14 (a procedure for the guideline updates). In “*Stakeholder involvement*” domain, target users in most CPGs were typically well-defined. Moreover, these guidelines provided details pertaining to the characteristics of the development panel members, including their names, professions, and institutional affiliations. However, few CPGs tried to seek the views and preferences of the target population through reasonable strategies, and detail this information.

#### 3.3.2 Reporting quality of CPGs

**Figure 3:**
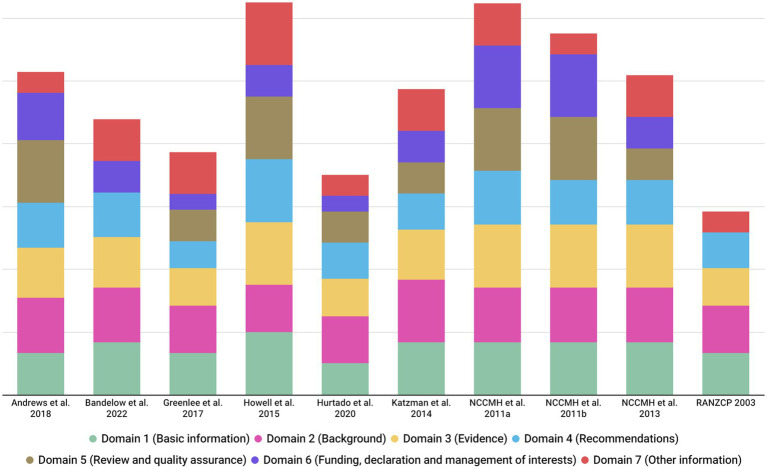
Global *RIGHT* scores by domain across 10 clinical practice guidelines.

**Figure 4:**
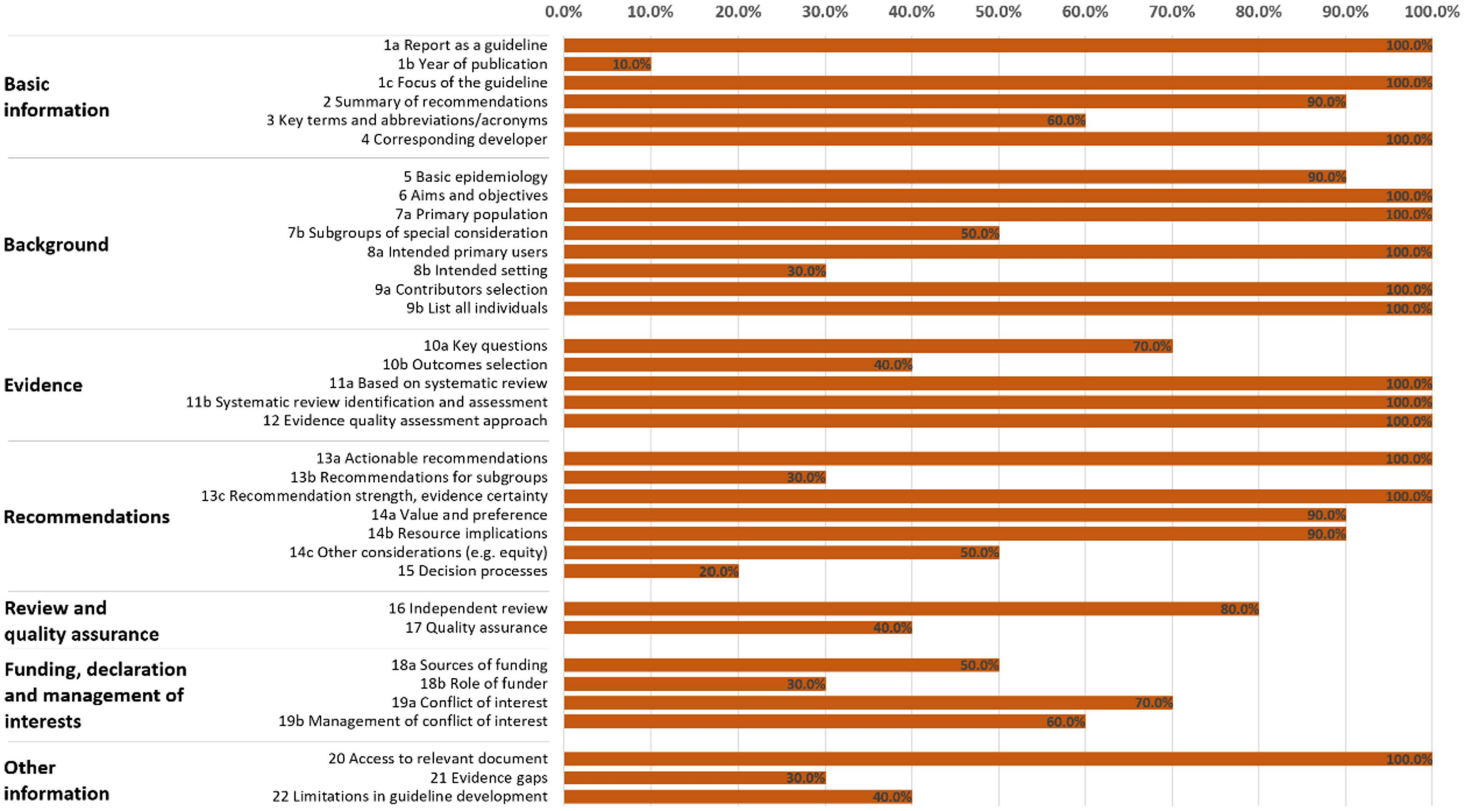
Overall reporting rate by *RIGHT* items across 10 clinical practice guidelines.

As shown in Figures 3, 4, and Appendix 7, the overall reporting rate of the ten included CPGs ranged from 51.4 to 88.6%. Thirty percent of the guidelines had an overall reporting rate higher than 80.0%.

Of the seven domains, the three with the highest reporting rates were, in descending order, “*Background*” (83.8%), “*Evidence*” (82.0%) and “*Basic information*” (76.7%) domain. The “*Funding, declaration and management of interests*” domain received the lowest reporting rate (52.5%). Six items had significant reporting defects (reporting rate ≤ 30%), namely 1b (year of publication; 10.0%), 8b (intended settings of the CPG; 30.0%), 13b (separate recommendations for subgroups if there are significant differences in factors influencing recommendations in the balance between benefits and harms across subgroups; 30%), 15 (processes and methods used by the CPG development panel to make decisions; 20.0%), 18b (role of funders in the different stages of CPG development and in the recommendations dissemination/implementation; 30%), and 21 (gaps in the current evidence and/or direct suggestions for future research; 30.0%). Fourteen items (i.e., 1a, 1c, 4, 6, 7a, 8a, 9a, 9b, 11a, 11b, 12, 13a, 13c and 20) were completely reported in all reviewed guidelines.

#### 3.3.3 Overall quality of CPGs

**Figure 5:**
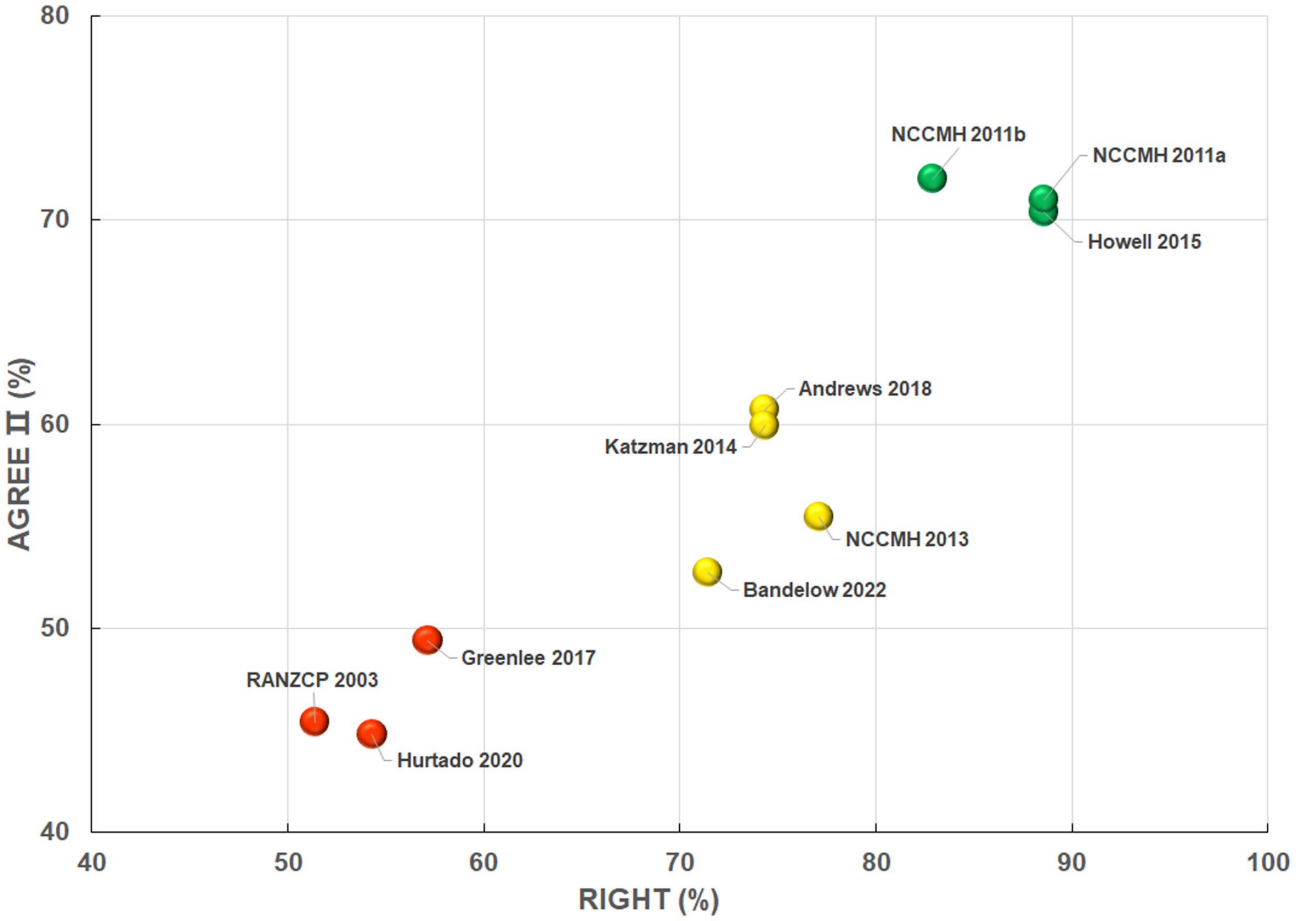
Grading and analysis of overall quality across 10 clinical practice guidelines.

According to the bubble plot, three CPGs (46, 47, 49) were classified as high-quality guidelines and could be recommended, three guidelines (45, 51, 52) were classified as low-quality guidelines and should not to be recommended, and the remaining four CPGs were classified as moderate-quality guidelines and required modification before being recommended (Figure 5). Overall, the trends in *RIGHT* checklist scores and *AGREE II* instrument scores were consistent, that is guidelines with better reporting completeness tended to have better methodological quality, and *vice versa*.

### 3.4 Recommendations of CAM

**Figure 6:**
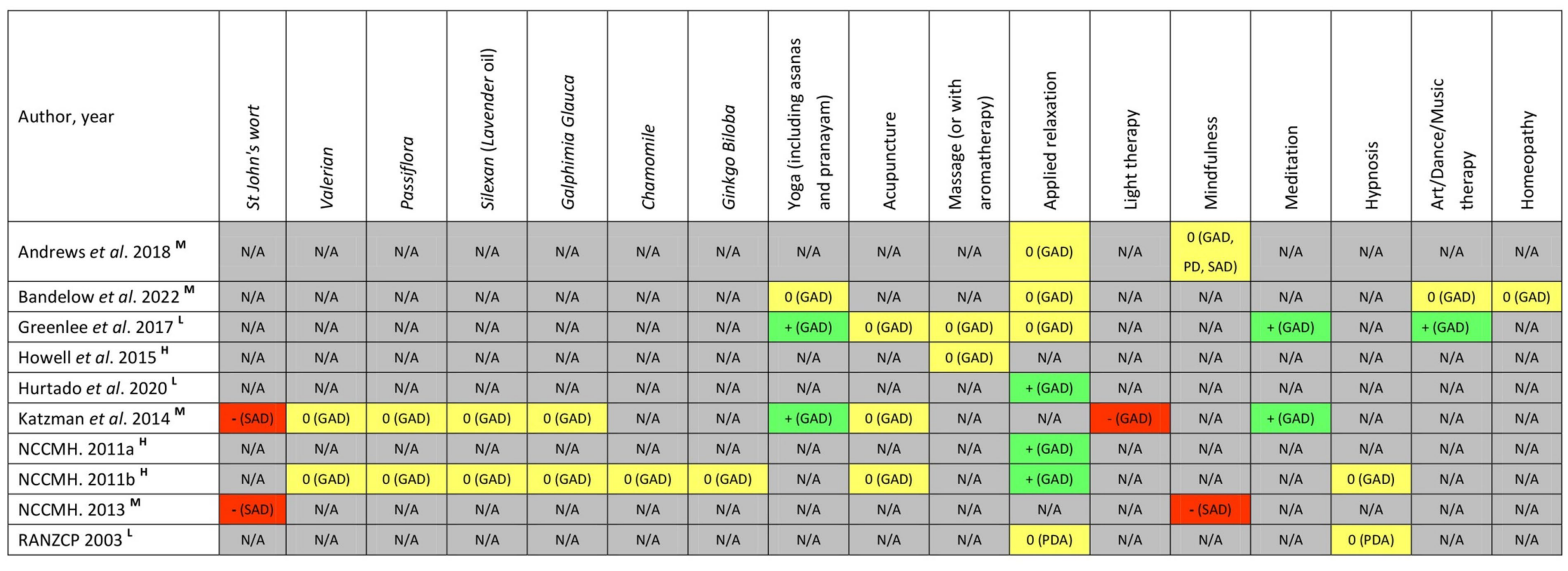
Summary of CAM recommendations in each clinical practice guideline. +/green = recommendations supporting the therapy/product use; −/red = recommendations against the therapy/product use; 0/yellow = recommendations unclear, uncertain, conflic ting, or “neither for nor against”; N/A/grey = no recommendations provided. The quality of CPGs is assessed according to *AGREE II* instrument and *RIGHT* checklist. H stands for high-quality CPG, M stands for moderate-quality CPG, L stands for low-quality CPG. GAD, generalised anxiety disorder; PD, panic disorder; PDA, panic disorder with agoraphobia; SAD, social anxiety disorder.

A summary of CAM recommendations for anxiety management across ten included CPGs is presented in Figure 6. Most of the CAM recommendations were provided for generalised anxiety disorder; a small number of recommendations were provided for social anxiety disorder, panic disorder, or panic disorder with agoraphobia. No specific CAM recommendations for separation anxiety disorder, specific phobia and selective mutism were identified in any reviewed CPGs.

Two CPGs strongly recommended meditation for the relief of generalised anxiety disorder (45, 50). Although yoga (45, 50), applied relaxation (46, 47, 51), and art/dance/music therapy (45) were also positively recommended in a few guidelines, they were considered to be recommended with caution in more guidelines due to the insufficient high-quality evidence of their effectiveness. Six phytomedicines, including *Valerian*, *Passiflora*, *Silexan* (*Lavender oil*), *Galphimia Glauca*, *Chamomile*, and *Ginkgo Biloba* have been used for the treatment of generalised anxiety disorder in clinical settings. Similarly, due to inadequate reliable evidence, these phytotherapeutics were marked as “neither for nor against” in the guidelines. Therapies that were defined as “uncertain recommendation” or “not recommended” for the same reason were acupuncture, massage, mindfulness, hypnosis, light therapy and homeopathy.

In the reviewed CPGs, none of the CAM therapies were recommended for the management of social anxiety disorder. Instead, two therapies [mindfulness (43, 48) and *St John’s wort* (48, 50)] were explicitly recommended not to be used due to questions about efficacy and concerns about safety, respectively.

There have been some reports regarding using applied relaxation, mindfulness or hypnosis for treating panic disorder with/without agoraphobia. Unfortunately, due to the lack of reliable evidence, no guidelines provided a definitive recommendation acknowledging the potential and benefits of any of these therapies (43, 52).

In general, only a few logically explained graded CAM recommendations were identified. Although the CPGs acknowledged that some CAM therapies might have potential benefits, the original studies underlying this evidence were however methodologically poor (as noted by the authors of the meta-analyses). Therefore, it was difficult to reach clear and unambiguous conclusions for or against the specific CAM use. Furthermore, none of the CPGs explicitly advise healthcare professionals to ask patients about their CAM use during anxiety and document such conditions within medical records.

## 4 Discussion

### 4.1 Summary of findings

In the included CPGs for anxiety management, CAM recommendations were distributed across 17 therapies or products. Of these, seven CAM modalities were phytotherapeutics; the rest involved mind–body practice, art therapy, and homeopathy. The recommendations target generalised anxiety disorder, social anxiety disorder and panic disorder with/without agoraphobia. However, most recommendations were unclear, uncertain, or “neither for nor against”. There were even recommendations that varied considerably between guidelines, often with conflicting information. This can result in variations in healthcare provision, and represents a gap in professional guidance that is particularly relevant in clinical practice (27). Explicitly graded recommendations supporting the CAM use were scarce, and all these recommendations were with respect to generalised anxiety disorder.

Sixty percent of the CPGs provided recommendations for the applied relaxation in the management of generalised anxiety disorder. Of these, high quality guidelines support its use as an effective option for anxiety relief. Light therapy was explicitly recommended not to be used for generalised anxiety disorder. While using mindfulness for social anxiety disorder remained controversial among different CPGs, *St. John’s wort* has been strongly advised against in the treatment of social anxiety disorder due to concerns about its potential interactions with prescribed as well as over-the counter medication (48). The recommendations for using applied relaxation, mindfulness or hypnosis in treating panic disorder with/without agoraphobia were unclear, given the existing evidence was insufficient in quantity and/or quality.

The overall reporting quality of the ten included CPGs was moderate to high (reporting rate from 51.4 to 88.6%). Of all these CPGs, seven were further rated as moderate to high in methodological quality.

Taken together, the published comprehensive guidelines are generally limited in incorporating clearly graded CAM recommendations. Furthermore, they are conservative and cautious about the application of CAM approaches in anxiety management.

### 4.2 Strengths, limitations, and comparison with previous systematic reviews

A previous systematic review within the same theme was published in 2022 (53). However, that review only included six CPGs (published from 2009 to 2020) covering five CAM modalities and did not perform reporting quality assessment. Also, the identified CAM recommendations did not correspond to the different types of anxiety disorders. Our review includes more up-to-date and eligible guidelines with more therapies, and adopted *RIGHT* checklist to appraise reporting quality of each CPG. We further listed the CAM therapies that are indicated or contraindicated for different anxiety disorders. Additionally, in that 2022 review, the methodological quality of CPGs was assessed by only two evaluators. In our review, four experienced evaluators conducted the standalone appraisal as recommended by the *AGREE II* instruction manual, and the *ICC* statistics showed good *IRR* across them. This allowed for a more comprehensive and unbiased conclusion. The quality of our review is further enhanced by the strong academic background of the researchers and multidisciplinary collaboration. The researchers who performed data extraction, quality appraisal, and outcome analysis had backgrounds in CAM, psychiatry, nursing science, clinical psychology, and/or evidence-based medicine, ensuring the reliability of the current reviewed results.

Some limitations of this review should be acknowledged. Many eligible guidelines may not have been captured based on our English/Chinese-only eligibility criteria. Given many traditional medicine systems originate from and integrated into national healthcare delivery systems in non-English/Chinese-speaking regions, such as India, Korea, Arab states, and Iran, CAM recommendations may be more prevalent in CPGs published in the official languages of these regions. In addition, both *AGREE II* instrument and *RIGHT* checklist were adopted to determine the quality of the overall guidelines rather than the CAM section of each guideline. We had to utilise the quality of the overall CPG to infer the quality and reliability of the CAM recommendations in each CPG. This is indirect rather than direct evidence. To inform healthcare providers with more applicable CAM recommendations in the anxiety management, future guidelines should incorporate broader, high-quality and rigorous CAM evidence while ensuring methodological and reporting quality. Finally, due to the NCCIH’s CAM classification criteria used in the current study, applied relaxation, meditation and mindfulness-related recommendations were included for analysis. However, it is necessary to acknowledge that these three modalities, namely applied relaxation (54), meditation (55), and mindfulness (56) have also been studied as either stand-alone psychotherapies or components of a standard treatment package for anxiety disorders.

### 4.3 Interpretation of the current findings

CAM is estimated to be used by more than 80% of the world’s population (57). Among population with psychiatric disorders, the use of CAM ranges from 20 to 80% (58). Including CAM in comprehensive CPGs therefore has significant relevance, including raising awareness of CAM among orthodox healthcare providers, helping improve patient access to CAM services, and encouraging more integrated care provision (59). However, none of the reviewed CPGs included recommendations enquiring about and/or documenting CAM use. Moreover, we only identified a few explicitly graded CAM recommendations from moderate to high quality CPGs to allow evidence-based communication and decision-making between clinical professionals and their customers in the treatment and care of generalised anxiety disorder and social anxiety disorder. Seventy percent of CPGs stated that low-quality and/or contradictory evidence from meta-analyses or original trials hindered the formation of credible CAM recommendations (43–45, 47, 49, 50, 52). The scarcity of CAM recommendations can also be attributed to other factors which impact the availability of CAM research, including general underestimation or biases against CAM therapies such as acupuncture (60) among mainstream medical community, and a lack of dedicated funding for CAM (61). Such a status quo may lead to the underuse of beneficial CAM approaches (61), especially in the context of lower risk of harms compared to other treatment options.

Data from a global epidemiological survey showed that more than 20.4 to 29.2% of patients with specific phobia visited mental health, general medical care, or CAM service for symptoms relief (62). A previous review suggested that some CAM and self-help therapies (e.g., bibliotherapy, massage, and relaxation training, etc.) may be helpful for anxiety commonly experienced in childhood, such as school phobia and separation anxiety (63). However, no reviewed guidelines included CAM recommendations for separation anxiety disorder, specific phobia or selective mutism (Figure 6). This represents a great missed opportunity to invite patients with these types of anxiety disorders to participate in shared decision-making about appropriate use of CAM, and to provide person-centered care where there is a known benefit (27).

Guidelines without credible CAM recommendations can also lead to the continued utilisation of potentially harmful CAM therapies (61). It is associated with the safety challenges in clinical practice, especially with respect to drug-induced liver injury (DILI) (64). After all, over-the-counter natural products have been the “main force” of CAM options in anxiety treatment. Of the 17 CAM modalities covered in this review, seven were phytotherapeutics (i.e., *St. John’s wort*, *Vlerian*, *Passiflora*, *Silexan*, *Galphimia Glauca*, *Chamomile*, and *Ginkgo Biloba*) (Figure 6). It is essential to point out that “naturalness” is not a guarantee of harmlessness, and that any pharmacologically active product is likely to have adverse effects (65). As one among the major causes for hepatotoxicity, CAM-related liver injury has a particularly high incidence in Asian countries where oriental medicine is popular (64). Data from Korea indicated that medicinal plants, poly-herbal preparations, and dietary supplements, were found to cause DILI in 9.4, 3.2, and 13.7% of patients, respectively (66). Traditional and complementary medicines account for 14.0% of the published data on DILI in India (67). In Japan, 7.1 and 10.0% of DILI were reported to be attributable to Chinese herb drugs and dietary supplements, respectively (68). Given the widespread use of Chinese medicines nationwide, China provides more reliable data based on a larger sample in the investigation of medicinal herb-induced liver damage. In two systematic analyses covering 9,335 and 24,112 patients with DILI respectively, Chinese medicines caused DILI in 18.6 and 21.2% cases (69). Another safety concern associated with the use of phytomedicine is the risk of interactions with prescribed pharmaceuticals. *St. John’s wort* was demonstrated to decrease plasma levels of benzodiazepines in healthy volunteers, and cause central serotonin syndrome by interacting with serotonin reuptake inhibitors (70); *Valerian* and *Passionflower* might increase the inhibitory activity of benzodiazepines binding to gamma-aminobutyric acid receptors, resulting in severe secondary effects (e.g., strong handshaking, palpitations, and dizziness, etc.) (71). As reported, while 55.3% of Australians have used prescribed pharmaceuticals to struggle with anxiety symptoms, 27.5% have used herbal medicines concurrently with prescribed pharmaceuticals (26). The lack of clear CAM recommendations therefore may cause clinicians to miss opportunity to guide patients to avoid risks from drug-herb interactions.

### 4.4 Implications for guideline development/updates and CAM clinical practice

#### 4.4.1 Implications for guideline development/updates

Guidelines need to be created using the most rigorous methodology to bridge the gap between research evidence and clinical practice (72). Three guidelines had significant flaws in methodology and completeness (Figure 5). Adoption of such guidelines usually results in difficulties with standardization and adaptation of care in resource-limited settings (73), waste of medical resources (40), and even harm to patients (74). While the overall quality of the remaining seven reviewed guidelines reached acceptable levels, there is still much room for improvement. It is suggested that in future updates, guidelines assigned low scores in individual or overall domains should be optimized based on the specifics highlighted in the *AGREE II* instrument (37) and *RIGHT* checklist (38), along with other guideline-related frameworks and checklists, such as *Institute of Medicine (US) Committee Criteria* (75), *GIN-McMaster Guideline Development Checklist* (76), and *PANELVIEW tool* (77). All CPGs included in this review received lowest scores in the “*Applicability*” domain of *AGREE II* instrument. Other studies showed consistent results (72). Low applicability reduces rate of use of guideline in daily clinical practice and prevents maximizing its positive impact on healthcare (72). A review of physician adherence to guidelines revealed that up to 38% of physicians considered guidelines as inconvenient or too difficult to use (78). The reporting completeness of the reviewed guidelines was generally unsatisfactory in the “Evidence to decision processes” domain of *RIGHT* checklist as well. For future anxiety guidelines, more attention should be paid to the basis of recommendations (i.e., values and preferences of the target population, cost, as well as the equity, feasibility and acceptability) and its application attribute (i.e., facilitators and barriers to CPG’s application, as well as advice, tools and potential resource implications on transferring the recommendations into practice).

As mentioned in the “*Limitation*” section, our judgment on the quality of CAM recommendations in existing CPGs was estimated based on a quality appraisal of the entire guideline rather than the CAM section of the CPG. Therefore, a strong requirement exists to develop a valid, reliable and practical tool that can be applied to the preparation and quality assessment of CAM recommendations in comprehensive guidelines. Elements such as clinical applicability, clarity, reliability/reproducibility, validity, clinical flexibility, and multidisciplinary process should be considered when developing such instrumentation (79).

Keeping guidelines updated is another challenge, as each step of searching for, synthesising, and appraising the evidence in order to make a graded recommendation is labor-intensive, time-consuming, and costly. Whereas, this process ensures that the up-to-date evidence is translated into accessible health outcomes in a timely manner (40). For CAM that mainstream clinicians do not specialize, it is even more critical to keep reflective of the sheer volume of the latest evidence in the guidelines.

We also notice that half of the included CPGs were developed by the medical societies/associations (43–45, 49, 52). Guidelines compiled by medical societies have been found to be often limited in quality (72). This can be attributed to medical societies/associations having a less diverse development panel consisting mainly of physicians. The perspective of other healthcare professionals and community members are necessary to improve the quality of certain domains of a guideline and its implementability (72). For guideline with a CAM component, it is pivotal to assemble a multidisciplinary development panel, including physicians/registered nurses, public health professionals, methodologists, editors, health policy makers/administrators, CAM practitioners with specialized expertise, health economist, and consumer representatives, rather than a group of physicians with mainstream medicine background only (42). Such stakeholder engagement, especially with diverse groups of end-users, allows for an evidence-based, transparent, and systematic approach to developing guidelines that are relevant and fit for purpose (27). These details have not gained the attention they deserve in the currently reviewed guidelines, which may result in a challenge of mismatch between provided recommendations and clinical practice. In an investigation performed in UK, 223 CAM organizations were enquired “*Which complementary and alternative therapies benefit which conditions*?” The answers showed that the top eight therapies advocated by professional CAM practitioners for the treatment of anxiety were aromatherapy, *Bach Flower*, hypnotherapy, massage, nutrition, reflexology, reiki, and yoga (80). From the patient’s perspective, NCCIH data suggested that the three most used CAM modalities are *Kava*, meditation, and relaxation-mental imagery (81). However, *Bach Flower*, *Kava*, nutrition, reflexology, and reiki were not included in any available guidelines we reviewed; meditation was mentioned in only two low-to-moderate quality guidelines and was only supported for relieving generalised anxiety disorder (Figure 6). Spiritual healing, biofeedback, *Echinacea*, and *Ginseng* are also widely used by patients with anxiety disorders (shown in the NCCIH survey) (81) but not incorporated in current CPGs. These findings serve as a reminder to guideline developers (and users) of the full consideration of patients’ preference and CAM practitioners’ advice when compiling (and implementing) clinical recommendations (and decisions).

Because of a lack of clear description of the systematic literature searches, one CPG (82) focusing on the treatment of dental anxiety was excluded in literature screening stage (Appendix 4). Effective management of dental anxiety and dental phobia is necessary given patients with these problems are candidates for syncope attacks in a dental chair (83). In a survey involving 320 dental patients, 68.8% of respondents reported using at least one CAM therapy for symptomatic relief (84). Similarly, CAM therapies (e.g., aromatherapy, massage, and music therapy, etc.) have been integrated into the nursing care to reduce anxiety and pain among laboring women in the United States (85). None of the currently reviewed guidelines addressed preoperative anxiety (including dental anxiety) and labor anxiety. Instead, 80% of guidelines provided recommendations for generalised anxiety disorder, and many of these recommendations overlapped. Such excessive duplication can create confusion for clinicians in the appropriate decision-making, and cause a waste of workload funding and other resources (86). These findings urge guideline developers to further improve the quality of their products. Integrating the efforts, expertise, and resources of multiple organizations through international networks or collaboration may help increase the efficiency of this process (72). To reduce the number of redundant or duplicative CPGs and increase the transparency of the development process, prospective registration of CPGs on a public registration platform for guidelines, such as PREPARE^1^ and GIN,^2^ is needed (87).

#### 4.4.2 Implications for CAM clinical practice

As clarified in a previous study, the quality rating score of a guideline cannot represent how it had affected clinical practice in the years following its publication (40). Guidelines graded as “recommended with modification” or “not recommended” in the bubble plot only refer to deficiencies in their development process and reporting information. It should not be misinterpreted that the CAM recommendations comprised in these guidelines are with lower and weaker clinical practice value (40). We, therefore, suggest that in clinical practice CAM recommendations in high-quality guidelines be positively considered but be carefully implemented in combination with specific clinical settings; while CAM recommendations in low-quality guidelines not be repudiated outright, but be withheld for the time being and determined once more high-quality evidence is accumulated. In addition, attention should be paid to the timeliness of guidelines (40). Given the publication date of the ten CPGs included in the current study spans 20 years (2003–2022), the recommendations summarized in Figure 6 might not reflect most up-to-date evidence well. Therefore, such CAM recommendations derived from earlier guidelines should be used with particular caution in the clinical settings.

Current evidence of the effectiveness and safety associated with CAM is mixed, with some approaches remaining controversial (88). Therefore, it is necessary to initiate the dialogue about CAM during medical consultations to minimize CAM risk and increase patient satisfaction (89). The reality, however, is that there is evidence indicating a high rate of non-disclosure of CAM application (27). For a variety of reasons (e.g., beliefs that their CAM use is not relevant to the physicians or physicians lack relevant knowledge, fear of discrimination, etc.), a considerable proportion of CAM users self- prescribe, rely on internet or advice from family and friends to guide their CAM decisions, and/or do not inform physicians about their CAM use (88). On the other hand, physicians rarely proactively ask patients about their use of CAM as well. The general knowledge gap makes most physicians uncomfortable when discussing CAM with their patients (88). Such neglect of discussion of CAM during medical visits may pose lots of potential medical risks, such as side effects of the herbs or drug-herb interaction that we mentioned earlier (88). Refining the CPG may be one way to reduce such risks. Guidelines are employed by mainstream healthcare professionals to inform practice decisions in unfamiliar fields such as CAM (61). Therefore, it is suggested that future updates to the guidelines emphasize that physicians must enquire about and document patients’ CAM use for anxiety management, thus triggering dialogue in the clinical settings and forging a better therapeutic alliance.

Although available guidelines have provided recommendations for 17 CAM modalities, there are still some other therapies which also showed potential in anxiety management that have not been documented in recommendations. These therapies, included but were not limited to, pharmacological/non-pharmacological approaches in Ayurveda [e.g., *Sankhapuspi* (*Convolvulus pluricaulis*), *Brahmi* (*Bacopa monnieri*), and *Ashwagandha* (*Withania somnifera*), etc.] (90), spiritual and religious interventions (e.g., prayer, religious meditation, and spiritual connection techniques, etc.) (91), and guided imagery (92). It is suggested that future updated CPGs collect evidence about the efficacy of these therapies, determine the evidence quality, and thus provide recommendations accordingly.

## 5 Conclusion

Despite a high percentage of anxiety patients who use CAM, the lack of recommendations, from available CPGs, for clinicians to enquire about and document CAM use in anxiety represents a major missed opportunity for shared decision-making. Guidelines were also conservative and cautious in recommending the application of CAM therapies. Inadequate high-quality clinical evidence and a lack of multidisciplinary development panel possibly underlie this position. The only consistent recommendations were: (1) meditation and applied relaxation were recommended for generalised anxiety disorder with light therapy not recommended; and (2) *St John’s wort* and mindfulness were not recommended for social anxiety disorder. To avoid the continued utilisation of potentially harmful CAM therapies, and/or the underuse of beneficial CAM therapies, more stringently designed trials are required to produce high-quality evidence and facilitate guidelines to formulate clear (pro or con) recommendations for each CAM modality. Various stakeholders should engage in the development of CPGs. In addition, there is an urgent need to develop a tool to determine the quality of CAM sections in the comprehensive CPGs. The *AGREE II* instrument and the *RIGHT checklist* should be used in future efforts to improve the overall quality of CPGs.

## Data availability statement

The original contributions presented in the study are included in the article/Supplementary material, further inquiries can be directed to the corresponding authors.

## Author contributions

F-YZ: Conceptualization, Formal analysis, Investigation, Methodology, Writing – original draft. GK: Conceptualization, Supervision, Writing – review & editing. PX: Data curation, Investigation, Writing – original draft. RC: Supervision, Writing – review & editing. Y-MW: Data curation, Funding acquisition, Software, Writing – review & editing. W-JZ: Funding acquisition, Methodology, Writing – review & editing. H-RW: Funding acquisition, Writing – review & editing. L-PY: Data curation, Software, Writing – review & editing. Y-LH: Methodology, Writing – review & editing. YW: Methodology, Writing – review & editing. YX: Investigation, Supervision, Writing – review & editing. Q-QF: Formal analysis, Methodology, Visualization, Writing – review & editing. ZZ: Project administration, Supervision, Writing – review & editing.

## Footnotes

^1^http://www.guidelines-registry.org/

^2^https://g-i-n.net/international-guidelines-library
