# Supplementary material for: Identifying complementary and alternative medicine recommendations for anxiety treatment and care: a systematic review and critical assessment of comprehensive clinical practice guidelines
Source: Front Psychiatry. 2023 Dec 12;14:1290580. doi: 10.3389/fpsyt.2023.1290580 (PMC10751921; doi:10.3389/fpsyt.2023.1290580)
Supplement: Supplementary file 1 [file Data_Sheet_1.pdf]

Appendices

Appendix 1 Categories of CAM modalities

Nutritional Approaches	Psychological Approaches	Physical Approaches	Combination Approaches		Other Complementary Health Approaches
			**Psychological and Physical Therapies (Mind and Body Practices**	**Psychological and Nutritional Therapies**	
• Special diets<br>• Dietary supplement<br>• Dietary plants<br>• Vitamins and minerals<br>• Botanical drugs<br>• Probiotics<br>• Prebiotics<br>• Microbial-based therapies	• Meditation<br>• Hypnosis<br>• Spiritual practice<br>• Mindfulness-based stress reduction	• Heat/Cold<br>• Light/Electrical/Magnetic stimulation<br>• Manual therapies<br>• Massage<br>• Acupuncture<br>• Spinal manipulation<br>• Chiropractic manipulation<br>• Osteopathic manipulation<br>• Movement education	• Yoga<br>• Pilates<br>• Tai-chi<br>• Art therapies<br>• Dance therapies<br>• Music therapies<br>• Relaxation techniques (e.g., breathing exercises, guided imagery)<br>• Qi-gong<br>• Trager psychophysical integration	• Mindful eating	• Traditional healers<br>• Ayurvedic medicine<br>• Traditional Chinese medicine<br>• Homeopathy<br>• Naturopathy<br>• Functional medicine

Notes: classification by the *US National Centre for Complementary and Integrative Health* (NCCIH)
https://www.nccih.nih.gov/health/complementary-alternative-or-integrative-health-whats-in-a-name

Appendix 2 Search strategy for each database

MEDLINE (via PubMed)	#1	clinical practice guideline[Title/Abstract]
	#2	clinical practice guideline[MeSH Terms]
	#3	clinical guideline[Title/Abstract]
	#4	clinical guideline[MeSH Terms]
	#5	practice guideline[Title/Abstract]
	#6	practice guideline[MeSH Terms]
	#7	guideline[Title/Abstract]
	#8	guideline[MeSH Terms]
	#9	guidance[Title/Abstract]
	#10	guidance[MeSH Terms] - Schema: all
	#11	recommendation[Title/Abstract]
	#12	recommendation[MeSH Terms] - Schema: all
	#13	consensus[Title/Abstract]
	#14	consensus[MeSH Terms]
	#15	expert consensus[Title/Abstract]
	#16	expert consensus[MeSH Terms]
	#17	statement[Title/Abstract]
	#18	statement[MeSH Terms] - Schema: all
	#19	#1 or #2 or #3 or #4 or #5 or #6 or #7 or #8 or #9 or #10 or #11 or #12 or #13 or #14 or #15 or #16 or #17 or #18
	#20	anxiety[Title/Abstract]
	#21	anxiety[MeSH Terms]
	#22	anxiety disorder[Title/Abstract]
	#23	anxiety disorder[MeSH Terms]
	#24	generalised anxiety disorder[Title/Abstract]
	#25	generalised anxiety disorder[MeSH Terms]
	#26	separation anxiety disorder[Title/Abstract]
	#27	separation anxiety disorder[MeSH Terms]
	#28	social anxiety disorder[Title/Abstract]
	#29	social anxiety disorder[MeSH Terms]
	#30	panic disorder[Title/Abstract]
	#31	panic disorder[MeSH Terms]
	#32	specific phobia[Title/Abstract]
	#33	specific phobia[MeSH Terms]
	#34	agoraphobia[Title/Abstract]
	#35	agoraphobia[MeSH Terms]
	#36	selective mutism[Title/Abstract]
	#37	selective mutism[MeSH Terms]
	#38	#20 or #21 or #22 or #23 or #24 or #25 or #26 or #27 or #28 or #29 or #30 or #32 or #32 or #33 or #34 or #35 or #36 or #37
	#39	#19 and #38
EMBASE (via OVID)	#1	'clinical practice guideline':ti,ab,kw

	#2 'clinical guideline':ti,ab,kw<br>#3 'practice guideline':ti,ab,kw<br>#4 'guidance':ti,ab,kw<br>#5 'recommendation':ti,ab,kw<br>#6 'consensus':ti,ab,kw<br>#7 'expert consensus':ti,ab,kw<br>#8 'statement':ti,ab,kw<br>#9 #1 OR #2 OR #3 OR #4 OR #5 OR #6 OR #7 OR #8<br>#10 'anxiety':ti,ab,kw<br>#11 'generalised anxiety disorder':ti,ab,kw<br>#12 'separation anxiety disorder':ti,ab,kw<br>#13 'social anxiety disorder':ti,ab,kw<br>#14 'panic disorder':ti,ab,kw<br>#15 'specific phobia':ti,ab,kw<br>#16 'agoraphobia':ti,ab,kw<br>#17 'selective mutism':ti,ab,kw<br>#18 #10 OR #11 OR #12 OR #13 OR #14 OR #15 OR #16 OR #17<br>#19 #9 AND #18
AMED: Allied and Complementary Medicine Database	#1 TI clinical practice guideline OR KW clinical practice guideline OR AB clinical practice guideline<br>#2 TI clinical guideline OR KW clinical guideline OR AB clinical guideline<br>#3 TI practice guideline OR KW practice guideline OR AB practice guideline<br>#4 TI guideline OR KW guideline OR AB guideline<br>#5 TI guidance OR KW guidance OR AB guidance<br>#6 TI recommendation OR KW recommendation OR AB recommendation<br>#7 TI consensus OR KW consensus OR AB consensus<br>#8 TI expert consensus OR KW expert consensus OR AB expert consensus<br>#9 TI statement OR KW statement OR AB statement<br>#10 S1 OR S2 OR S3 OR S4 OR S5 OR S6 OR S7 OR S8 OR S9<br>#11 TI anxiety OR KW anxiety OR AB anxiety<br>#12 TI anxiety disorder OR KW anxiety disorder OR AB anxiety disorder<br>#13 TI generalised anxiety disorder OR KW generalised anxiety disorder OR AB generalised anxiety disorder<br>#14 TI separation anxiety disorder OR KW separation anxiety disorder OR AB separation anxiety disorder<br>#15 TI social anxiety disorder OR KW social anxiety disorder OR AB social anxiety disorder<br>#16 TI panic disorder OR KW panic disorder OR AB panic disorder<br>#17 TI specific phobia OR KW specific phobia OR AB specific phobia<br>#18 TI agoraphobia OR KW agoraphobia OR AB agoraphobia<br>#19 TI selective mutism OR KW selective mutism OR AB selective mutism<br>#20 #11 OR #12 OR #13 OR #14 OR #15 OR #16 OR #17 OR #18 OR #19<br>#21 #10 AND #20
China National Knowledge Infrastructure (CNKI)	(篇关摘=临床实践指南) OR (篇关摘=临床指南) OR (篇关摘=指南) OR (篇关摘=共识) OR (篇关摘=声明) AND (篇关摘=焦虑症) OR (篇关摘=焦虑障碍) OR (篇关摘=广泛性焦虑障碍) OR (篇关摘=广场恐怖症) OR (篇关摘=惊恐发作) OR (篇关摘=惊恐障碍) OR (篇关摘=社交焦虑障碍) OR (篇关摘=社交恐怖症) OR (篇关摘=分离焦虑障碍) OR (篇关摘=选择性缄默症) OR (篇关摘=特定恐怖症)
Wanfang database	题名或关键词:(临床实践指南 or 临床指南 or 指南 or 共识 or 声明) and 题名或关键词:(焦虑症 or 焦虑障碍 or

	广泛性焦虑障碍 or 广场恐怖症 or 惊恐发作 or 惊恐障碍 or 社交焦虑障碍 or 社交恐怖症 or 分离焦虑障碍 or 选择性缄默症 or 特定恐怖症)
Chongqing VIP database (CQVIP)	题名或关键词=临床实践指南 OR 题名或关键词=临床指南 OR 题名或关键词=指南 OR 题名或关键词=共识 OR 题名或关键词=声明 AND 题名或关键词=失眠 OR 题名或关键词=焦虑症 OR 题名或关键词=焦虑障碍 OR 题名或关键词=广泛性焦虑障碍 OR 题名或关键词=广场恐怖症 OR 题名或关键词=惊恐发作 OR 题名或关键词=惊恐障碍 OR 题名或关键词=社交焦虑障碍 OR 题名或关键词=社交恐怖症 OR 题名或关键词=分离焦虑障碍 OR 题名或关键词=选择性缄默症 OR 题名或关键词=特定恐怖症
China biomedical literature service system (SinoMed)	"临床实践指南"[标题:智能] OR "临床指南"[标题:智能] OR "指南"[标题:智能] OR "共识"[标题:智能] OR "声明"[标题:智能] AND "焦虑症"[标题:智能] OR "焦虑障碍"[标题:智能] OR "广泛性焦虑障碍"[标题:智能] OR "广场恐怖症"[标题:智能] OR "惊恐发作"[标题:智能] OR "惊恐障碍"[标题:智能] OR "社交焦虑障碍"[标题:智能] OR "社交恐怖症"[标题:智能] OR "分离焦虑障碍"[标题:智能] OR "选择性缄默症"[标题:智能] OR "特定恐怖症"[标题:智能]
NCCIH website	https://www.nccih.nih.gov/health/providers/clinicalpractice
Guideline related websites	Guidelines International Network (https://g-i-n.net/)
	National Institute for Health and Clinical Excellence (https://www.nice.org.uk/)
	British Columbia guideline (http://www.bcguidelines.ca/alphabetica)
	National Guideline Clearinghouse (https://www.ahrq.gov/gam/index.html)
	Scottish Intercollegiate Guidelines Network (https://www.sign.ac.uk/)
	Canadian Medical Association: Clinical Practice Guideline (https://joulecma.ca/)

Appendix 3 *NCCIH* website and websites of six international guidelines developing institutions

Websites	URL	Number of documents retrieved
National Center for Complementary and Integrative Health (NCCIH)	https://www.nccih.nih.gov/health/providers/clinicalpractice	0
Guidelines International Network (GIN)	https://g-i-n.net/	21
British Columbia guideline (BC Guidelines)	http://www.bcguidelines.ca/alphabetica	393
National Guideline Clearinghouse (NGC)	https://www.ahrq.gov/gam/index.html	1390
Scottish Intercollegiate Guidelines Network (SIGN)	https://www.sign.ac.uk/	76
Canadian Medical Association: Clinical Practice Guideline (CMA-CPG Infobase)	https://joulecma.ca/	0
National Institute for Health and Clinical Excellence (NICE)	https://www.nice.org.uk/	305

Appendix 4 Excluded clinical practice guidelines with detailed reasons of irrelevance

References	Reasons for exclusion
Baldwin DS, Anderson IM, Nutt DJ, Bandelow B, Bond A, Davidson JR, den Boer JA, Fineberg NA, Knapp M, Scott J, Wittchen HU; British Association for Psychopharmacology. Evidence-based guidelines for the pharmacological treatment of anxiety disorders: recommendations from the British Association for Psychopharmacology. *J Psychopharmacol*, 2005; 19(6):567-596.	without CAM information
Bandelow B, Lichte T, Rudolf S, Wiltink J, Beutel ME. The diagnosis of and treatment recommendations for anxiety disorders. *Dtsch Arztebl Int*, 2014; 111(27-28):473-480.	earlier version of the included CPG
Bandelow B, Lichte T, Rudolf S, Wiltink J, Beutel ME. The German guidelines for the treatment of anxiety disorders. *Eur Arch Psychiatry Clin Neurosci*, 2015; 265(5):363-73.	earlier version of the included CPG
Bandelow B, Michaelis S, Wedekind D. Treatment of anxiety disorders. *Dialogues Clin Neurosci*, 2017; 19(2):93-107.	without a systematic literature searches
Bandelow B, Sher L, Bunevicius R, Hollander E, Kasper S, Zohar J, Möller HJ; WFSBP Task Force on Mental Disorders in Primary Care; WFSBP Task Force on Anxiety Disorders, OCD and PTSD. Guidelines for the pharmacological treatment of anxiety disorders, obsessive-compulsive disorder and posttraumatic stress disorder in primary care. *Int J Psychiatry Clin Pract*, 2012; 16(2):77-84.	without CAM information
Canadian Psychiatric Association. Clinical practice guidelines. Management of anxiety disorders. *Can J Psychiatry*, 2006; 51(8 Suppl 2):9S-91S.	earlier version of the included CPG
Chinese Medical Association (Psychiatry Branch). Prevention and Treatment Guidelines for Anxiety Disorders [article in Chinese]. People's Medical Publishing House, Beijing, China: 2010.	without a systematic literature searches
Chinese Medical Association, Journal agency of the Chinese Medical Association, General Practice Branch of Chinese Medical Association, Psychiatry Branch (Anxiety Disorders Collaborative Group) of the Chinese Medical Association, Editorial Committee of the Chinese Journal of General Practitioners, Expert group of Primary Care Guidelines for Neurological Disorders. Primary care guidelines for generalized anxiety disorder (2021) [article in Chinese]. *Chinese Journal of General Practitioners*, 2021; 20(12):1232-1241.	without a systematic literature searches
Connolly SD, Bernstein GA; Work Group on Quality Issues. Practice parameter for the assessment and treatment of children and adolescents with anxiety disorders. *J Am Acad Child Adolesc Psychiatry*, 2007; 46(2):267-283.	without CAM information
Creswell C, Waite P, Cooper PJ. Assessment and management of anxiety disorders in children and adolescents. *Arch Dis Child*, 2014; 99(7):674-678.	without CAM information
Dow SP, Sonies BC, Scheib D, Moss SE, Leonard HL. Practical guidelines for the assessment and treatment of selective mutism. *J Am Acad Child Adolesc Psychiatry*, 1995; 34(7):836-846.	without CAM information
Garland EJ, Kutcher S, Virani A. 2008 position paper on using SSRIs in children and adolescents. *J Can Acad Child Adolesc Psychiatry*, 2009; 18(2):160-165.	without CAM information
Gautam S, Jain A, Gautam M, Vahia VN, Gautam A. Clinical practice guidelines for the management of generalised anxiety disorder (GAD) and panic disorder (PD). *Indian J Psychiatry*, 2017; 59(Suppl 1):S67-S73.	without a systematic literature searches

Gautam S, Jain A, Marwale AV, Gautam A. Clinical practice guidelines for Yoga and other alternative therapies for patients with mental disorders. *Indian J Psychiatry*, 2020; 62(Suppl 2):S272-S279.	without a systematic literature searches
Gim M, Kim MK, Lee JH, Kim W, Moon E, Seo HJ, Koo BH, Yang JC, Lee KS, Lee SH, Kim CH, Yu BH, Suh HS. Korean guidelines for the treatment of panic disorder 2018: Psychosocial Treatment Strategies. *Anxiety and mood*, 2019; 15(1): 13-19.	not published in English or Chinese
Grassi L, Caruso R, Riba M B, Lloyd-Williams M, Kissane D, Rodin G, McFarland D, Campos-Ródenas R, Zachariae R, Santini D, Ripamonti CI. Anxiety and depression in adult cancer patients: ESMO Clinical Practice Guideline. *ESMO Open*, 2023.	without CAM information
Guang'an Men Hospital, China Academy of Chinese Medical Sciences. Individualized-based TCM clinical practice guidelines for generalized anxiety disorder [article in Chinese]. *World Journal of Sleep Medicine*, 2016; 3(2):80-94.	non-comprehensive CPGs (TCM specialized)
Guidelines and Protocols Advisory Committee. Anxiety and depression in children and youth-diagnosis and treatment. Victoria, BC: British Columbia Ministry of Health, 2010.	without CAM information
Gurney J, Onifer D, Pamplin J, Martin M, Fields A, William N, Tobin J, Stockinger Z, Hatzfeld JJ, Sonka B, Shackelford SA. Pain, Anxiety and Delirium (CPG ID: 29). Online available: https://jts.amedd.army.mil/assets/docs/cpgs/Pain_Anxiety_Delirium_26_Apr_2021_ID29.pdf	without CAM information
Howell D, Keller-Olaman S, Oliver TK, Hack TF, Broadfield L, Biggs K, Chung J, Gravelle D, Green E, Hamel M, Harth T, Johnston P, McLeod D, Swinton N, Syme A, Olson K. A pan-Canadian practice guideline and algorithm: screening, assessment, and supportive care of adults with cancer-related fatigue. *Curr Oncol*, 2013; 20(3): e233-246.	without CAM information
Keen DV, Fonseca S, Wintgens A. Selective mutism: a consensus based care pathway of good practice. *Archives of disease in childhood*, 2008; 93(10): 838-844.	without CAM information
Kim MK, Lee JH, Gim M, Kim W, Moon E, Seo HJ, Koo BH, Yang JC, Lee KS, Lee SH, Kim CH, Yu BH, Suh HS. Korean guidelines for the treatment of panic disorder 2018: initial and maintenance treatment strategies for the pharmacological treatment of panic disorder. *Anxiety and mood*, 2018; 14(2): 53-62.	not published in English or Chinese
Lim L, Chan HN, Chew PH, Chua SM, Ho C, Kwek SK, Lee TS, Loh P, Lum A, Tan YH, Wan YM, Woo M, Yap HL. Ministry of Health Clinical Practice Guidelines: Anxiety disorders. *Singapore Med J*, 2015; 56(6):310-315.	without CAM information
Lyndon B, Rowe L, Fraser A, Efron D, Walter G, Wilson I, Newman L, Silove N; Royal Australian and New Zealand College of Psychiatrists; Royal Australasian College of Physicians; Royal Australian College of General Practitioners. Clinical guidance on the use of antidepressant medications in children and adolescents. *Aust Fam Physician*, 2005; 34(9):777-778.	without CAM information
Mazza D, Chakraborty SP, Brijnath B, Nowak H, Howell C, Brott T, Atchison M, Gras D, Kenardy J, Buchanan R, Tawia S. Diagnosing and managing work-related mental health conditions in general practice: new Australian clinical practice guidelines. *Med J Aust*, 2019; 211(2):76-81.	without CAM information
NHS. Clinical guide for dental anxiety management. https://www.england.nhs.uk/long-read/clinical-guide-for-dental-anxiety-management/.	without a systematic literature searches
Oliveira J E Silva L, Prakken SD, Meltzer AC, Broder JS, Gerberi DJ, Upadhye S, Carpenter CR, Bellolio F. Depression and anxiety screening in emergency department patients with recurrent abdominal pain: An evidence synthesis for a clinical practice guideline. *Acad Emerg Med*, 2022; 29(5):615-629.	without CAM information

Raju NN, Naga Pavan Kumar KSVR, Nihal G. Clinical practice guidelines for assessment and management of anxiety and panic disorders in emergency setting. *Indian J Psychiatry*, 2023; 65(2):181-185.	without a systematic literature searches
Reddy YCJ, Sudhir PM, Manjula M, Arumugham SS, Narayanaswamy JC. Clinical practice guidelines for cognitive-behavioral therapies in anxiety disorders and obsessive-compulsive and related disorders. *Indian J Psychiatry*, 2020; 62(Suppl 2):S230-S250.	without a systematic literature searches
Roy-Byrne P, Stein M, Bystrisky A, Katon W. Pharmacotherapy of panic disorder: proposed guidelines for the family physician. *J Am Board Fam Pract*, 1998; 11(4):282-290.	without CAM information
Sarris J, Ravindran A, Yatham LN, Marx W, Rucklidge JJ, McIntyre RS, Akhondzadeh S, Benedetti F, Caneo C, Cramer H, Cribb L, de Manincor M, Dean O, Deslandes AC, Freeman MP, Gangadhar B, Harvey BH, Kasper S, Lake J, Lopresti A, Lu L, Metri NJ, Mischoulon D, Ng CH, Nishi D, Rahimi R, Seedat S, Sinclair J, Su KP, Zhang ZJ, Berk M. Clinician guidelines for the treatment of psychiatric disorders with nutraceuticals and phytoceuticals: The World Federation of Societies of Biological Psychiatry (WFSBP) and Canadian Network for Mood and Anxiety Treatments (CANMAT) Taskforce. *World J Biol Psychiatry*, 2022; 23(6):424-455.	non-comprehensive CPGs (nutraceuticals and phytoceuticals specialized)
Schaffer A, McIntosh D, Goldstein BI, Rector NA, McIntyre RS, Beaulieu S, Swinson R, Yatham LN; Canadian Network for Mood and Anxiety Treatments (CANMAT) Task Force. The CANMAT task force recommendations for the management of patients with mood disorders and comorbid anxiety disorders. *Ann Clin Psychiatry*, 2012; 24(1):6-22.	without CAM information
Seedat S. Social anxiety disorder (social phobia): The south African society of psychiatrists (SASOP) treatment guidelines for psychiatric disorders. *South African Journal of Psychiatry*, 2013, 19(3): 192-196.	without CAM information
Stein D J. Generalised anxiety disorder: The South African Society of Psychiatrists (SASOP) treatment guidelines for psychiatric disorders. *South African Journal of Psychiatry*, 2013; 19(3): 175-179.	without CAM information
Subramanyam AA, Kedare J, Singh OP, Pinto C. Clinical practice guidelines for geriatric anxiety disorders. *Indian J Psychiatry*, 2018; 60(Suppl 3):S371-S382.	without a systematic literature searches
Suh HS, Lee JH, Gim MS, Kim MK. Korean guidelines for the treatment of panic disorder. *Journal of the Korean Medical Association*, 2018; 61(8): 493-499.	not published in English or Chinese
Szabo CP. Panic disorder: The south African society of psychiatrists (SASOP) treatment guidelines for psychiatric disorders. *South African Journal of Psychiatry*, 2013; 19(3): 172-174.	without CAM information
Tang QS. International clinical practice guideline of Chinese medicine anxiety. *World J Tradit Chin Med*, 2021; 7:280-286.	non-comprehensive CPGs (TCM specialized)
Walter HJ, Bukstein OG, Abright AR, Keable H, Ramtekkar U, Ripperger-Suhler J, Rockhill C. Clinical practice guideline for the assessment and treatment of children and adolescents with anxiety disorders. *J Am Acad Child Adolesc Psychiatry*, 2020; 59(10):1107-1124.	without CAM information
World Federation of Chinese Medicine Societies, Chinese Society of Traditional Chinese Medicine. International TCM clinical practice guidelines for anxiety disorders (2020-10-11) [article in Chinese]. *World Chinese Medicine*, 2021; 16(8):1188-1191.	non-comprehensive CPGs (TCM specialized)

Abbreviations CPG(s), clinical practice guideline(s); CAM, complementary and alternative medicine; TCM, Traditional Chinese Medicine.

Appendix 5 Inter-rater reliability for each domain of the *AGREE II* instrument

**Domains**	***ICCs***	**95% *CI***		***p***
		Lower-bound	Upper-bound	
Scope and purpose	0.89	0.81	0.94	< 0.01
Stakeholder involvement	0.70	0.48	0.84	< 0.01
Rigor of development	0.88	0.83	0.92	< 0.01
Clarity of presentation	0.90	0.82	0.95	< 0.01
Applicability	0.80	0.67	0.89	< 0.01
Editorial independence	0.75	0.52	0.89	< 0.01
Overall	0.89	0.87	0.91	< 0.01

Abbreviations: *ICCs*, Intraclass Correlation Coefficients; *CI*, confidence intervals.

Appendix 6 Methodological quality of each clinical practice guideline appraised by the *AGREE II* instrument

Author, year	Six domains of AGREE II						Overall score	Overall quality
	Scope and purpose (%)	Stakeholder involvement (%)	Rigor of development (%)	Clarity of presentation (%)	Applicability (%)	Editorial independence (%)		
Andrews *et al*. 2018 (43)	75.0	54.2	66.7	68.1	33.3	66.7	60.7	M
Bandelow *et al*. 2022 (44)	66.7	48.6	53.1	70.8	20.8	56.3	52.7	M
Greenlee *et al*. 2017 (45)	69.4	48.6	58.3	59.7	29.2	31.3	49.4	L
Howell *et al*. 2015 (49)	86.1	83.3	73.4	86.1	33.3	60.4	70.4	H
Hurtado *et al*. 2020 (51)	51.4	58.3	65.6	44.4	17.7	31.3	44.8	L
Katzman *et al*. 2014 (50)	77.8	56.9	61.5	63.9	17.7	81.3	59.9	M
NCCMH *et al*. 2011a (46)	84.7	66.7	66.1	83.3	39.6	85.4	71.0	H
NCCMH *et al*. 2011b (47)	86.1	66.7	69.3	84.7	39.6	85.4	72.0	H
NCCMH *et al*. 2013 (48)	81.9	56.9	63.5	70.8	21.9	37.5	55.4	M
RANZCP 2003 (52)	62.5	61.1	50.0	66.7	21.9	10.4	45.4	L
Mean ± SD	74.2 ± 11.6	60.1 ± 10.3	62.8 ± 7.2	69.9 ± 12.8	27.5 ± 8.6	54.6 ± 26.1	58.2 ± 10.4	

Notes low quality, overall scores < 50%; moderate quality, 50% ≤ overall scores ≤ 70%; high quality, overall scores > 70%.

Abbreviations L, low quality; M, moderate quality; H, high quality.

Appendix 7 Reporting quality of each clinical practice guideline appraised by the *RIGHT* checklist

	Andrews *et al.* 2018 (43)	Bandelow *et al.* 2022 (44)	Greenlee *et al.* 2017 (45)	Howell *et al.* 2015 (49)	Hurtado *et al.* 2020 (51)	Katzman *et al.* 2014 (50)	NCCMH *et al.* 2011a (46)	NCCMH *et al.* 2011b (47)	NCCMH *et al.* 2013 (48)	RANZCP 2003 (52)
Domain 1: Basic information	4 (66.7)	5 (83.3)	4 (67.7)	6 (100.0)	3 (50.0)	5 (83.3)	5 (83.3)	5 (83.3)	5 (83.3)	4 (67.7)
Title/subtitle										
1a Report as a guideline	Y	Y	Y	Y	Y	Y	Y	Y	Y	Y
1b Year of publication	N	N	N	Y	N	N	N	N	N	N
1c Focus of the guideline	Y	Y	Y	Y	Y	Y	Y	Y	Y	Y
Executive summary										
2 Summary of recommendations	Y	Y	Y	Y	N	Y	Y	Y	Y	Y
Abbreviations and acronyms										
3 Key terms and abbreviations/acronyms	N	Y	N	Y	N	Y	Y	Y	Y	N
Corresponding developer										
4 Corresponding developer	Y	Y	Y	Y	Y	Y	Y	Y	Y	Y
Domain 2: Background	7 (87.5)	7 (87.5)	6 (75.0)	6 (75.0)	6 (75.0)	8 (100.0)	7 (87.5)	7 (87.5)	7 (87.5)	6 (75.0)
Brief description of the health problem(s)										
5 Basic epidemiology	Y	Y	Y	N	Y	Y	Y	Y	Y	Y
Aim(s) of the guideline and specific objectives										
6 Aims and objectives	Y	Y	Y	Y	Y	Y	Y	Y	Y	Y
Target populations										
7a Primary population	Y	Y	Y	Y	Y	Y	Y	Y	Y	Y
7b Subgroups of special consideration	Y	Y	N	Y	N	Y	N	N	Y	N
End users and settings										
8a Intended primary users	Y	Y	Y	Y	Y	Y	Y	Y	Y	Y

8b Intended setting	N	N	N	N	N	Y	Y	Y	N	N
Guideline development groups										
9a Contributors selection	Y	Y	Y	Y	Y	Y	Y	Y	Y	Y
9b List all individuals	Y	Y	Y	Y	Y	Y	Y	Y	Y	Y
Domain 3: Evidence	4 (80.0)	4 (80.0)	3 (60.0)	5 (100.0)	3 (60.0)	4 (80.0)	5 (100.0)	5 (100.0)	5 (100.0)	3 (60.0)
Health care questions										
10a Key questions	Y	Y	N	Y	N	Y	Y	Y	Y	N
10b Outcomes selection	N	N	N	Y	N	N	Y	Y	Y	N
Systematic reviews										
11a Based on systematic review	Y	Y	Y	Y	Y	Y	Y	Y	Y	Y
11b Systematic review identification and assessment	Y	Y	Y	Y	Y	Y	Y	Y	Y	Y
Assessment of the certainty of the body of evidence										
12 Evidence quality assessment approach	Y	Y	Y	Y	Y	Y	Y	Y	Y	Y
Domain 4: Recommendations	5 (71.4)	5 (71.4)	3 (42.9)	7 (100.0)	4 (57.1)	4 (57.1)	6 (85.7)	5 (71.4)	5 (71.4)	4 (57.1)
Recommendations										
13a Actionable recommendations	Y	Y	Y	Y	Y	Y	Y	Y	Y	Y
13b Recommendations for subgroups	N	N	N	Y	N	N	Y	N	Y	N
13c Recommendation strength, evidence certainty	Y	Y	Y	Y	Y	Y	Y	Y	Y	Y
Rationale/explanation for recommendations										
14a Value and preference	Y	Y	Y	Y	Y	Y	Y	Y	Y	N
14b Resource implications	Y	Y	N	Y	Y	Y	Y	Y	Y	Y
14c Other considerations (e.g. equity)	Y	N	N	Y	N	N	Y	Y	N	Y

Evidence to decision processes										
15 Decision processes	N	Y	N	Y	N	N	N	N	N	N
Domain 5: Review and quality assurance	2 (100.0)	0 (0.0)	1 (50.0)	2 (100.0)	1 (50.0)	1 (50.0)	2 (100.0)	2 (100.0)	1 (50.0)	0 (0.0)
External review										
16 Independent review	Y	N	Y	Y	Y	Y	Y	Y	Y	N
Quality assurance										
17 Quality assurance	Y	N	N	Y	N	N	Y	Y	N	N
Domain 6: Funding, declaration and management of interests	3 (75.0)	2 (50.0)	1 (25.0)	2 (50.0)	1 (25.0)	2 (50.0)	4 (100.0)	4 (100.0)	2 (50.0)	0 (0.0)
Funding source(s) and role(s) of the funder										
18a Sources of funding	Y	Y	N	N	N	Y	Y	Y	N	N
18b Role of funder	N	N	N	Y	N	N	Y	Y	N	N
Declaration and management of interest										
19a Conflict of interest	Y	N	Y	Y	Y	N	Y	Y	Y	N
19b Management of conflict of interest	Y	Y	N	N	N	Y	Y	Y	Y	N
Domain 7: Other information	1 (33.3)	2 (66.7)	2 (66.7)	3 (100.0)	1 (33.3)	2 (66.7)	2 (66.7)	1 (33.3)	2 (66.7)	1 (33.3)
Access										
20 Access to relevant document	Y	Y	Y	Y	Y	Y	Y	Y	Y	Y
Suggestions for further research										
21 Evidence gaps	N	Y	N	Y	N	Y	N	N	N	N
Limitations of the guideline										
22 Limitations in guideline development	N	N	Y	Y	N	N	Y	N	Y	N
**No. of reported items (%)**	26 (74.3)	25 (71.4)	20 (57.1)	31 (88.6)	19 (54.3)	26 (74.3)	31 (88.6)	29 (82.9)	27 (77.1)	18 (51.4)
